# Novel antimicrobial peptides against *Cutibacterium acnes* designed by deep learning

**DOI:** 10.1038/s41598-024-55205-3

**Published:** 2024-02-24

**Authors:** Qichang Dong, Shaohua Wang, Ying Miao, Heng Luo, Zuquan Weng, Lun Yu

**Affiliations:** 1 Shanghai MetaNovas Biotech Co., Ltd, Shanghai, 200120 China; 2 Metanovas Biotech Inc., Foster City, 94404 USA; 3 College of Biological Science and Engineering, Fuzhou University, Fuzhou, 350108 China

**Keywords:** Antimicrobial peptides, Deep learning, Transfer learning, *Cutibacterium acnes*, Pretrained protein language embedding, Biochemistry, Peptides, Virtual screening, Machine learning, Virtual drug screening

## Abstract

The increasing prevalence of antibiotic resistance in *Cutibacterium acnes* (*C. acnes*) requires the search for alternative therapeutic strategies. Antimicrobial peptides (AMPs) offer a promising avenue for the development of new treatments targeting *C. acnes*. In this study, to design peptides with the specific inhibitory activity against *C. acnes*, we employed a deep learning pipeline with generators and classifiers, using transfer learning and pretrained protein embeddings, trained on publicly available data. To enhance the training data specific to *C. acnes* inhibition, we constructed a phylogenetic tree. A panel of 42 novel generated linear peptides was then synthesized and experimentally evaluated for their antimicrobial selectivity and activity. Five of them demonstrated their high potency and selectivity against *C. acnes* with MIC of 2–4 µg/mL. Our findings highlight the potential of these designed peptides as promising candidates for anti-acne therapeutics and demonstrate the power of computational approaches for the rational design of targeted antimicrobial peptides.

## Introduction

Natural AMPs are a set of small proteins synthesized by microorganisms, plants, and animals as part of their host innate immune response to infection. They often show good activities against multi-drug resistant bacteria, thereby offering an opportunity to address this global public health threat^1,2^. Most reported AMPs are cationic and amphiphilic in nature and possess properties that are thought to be crucial for insertion into and disruption of the bacterial membrane^3,4^.

In this paper, we are concerned with *C. acnes*, formerly known as *Propionibacterium acnes*^5^, a gram-positive (GP) bacterium that colonizes human skin. This lipophilic anaerobic bacterium resides mainly in the sebum-rich pilosebaceous units but is also detected in non-sebaceous areas^6^. *C. acnes* play an important role in the pathophysiology of acne vulgaris^7^. Acne vulgaris is a chronic inflammatory skin disorder affecting more than 80% of all adolescents and young adults worldwide^8^. Topical antibiotics, such as clindamycin, are effective acne treatments, but their widespread and often permissive use has led to the generation of resistant strains^9^. Owing to their bioactivities and low tendency to induce resistance, new antimicrobial agents, specifically targeting biofilm-forming *C. acnes*, may represent potential treatments to modulate the skin microbiota in acne^10^.

Previous designs of peptides against *C. acnes* were mostly template-based, relying on natural peptide screening, derivation, and sequence modifications^7,11,12^. The success of these template-based designs is highly dependent on prior knowledge and predefined rules discovered from existing AMPs. However, identifying and experimentally validating these rules can be challenging, time-consuming and costly^13^.

With the progress of artificial intelligence, model-based methods have been applied to design AMPs. For the model-based de novo peptides design, two kinds of models are used: (1) generative models that generate novel peptide sequences; (2) predictive models that predict the bioactivities and properties of peptides by taking the peptide sequences as input. Various model-based methods have been used to design new AMPs with high antimicrobial activity, resistance to proteolysis, and low toxicity^14–24^, but only a limited of them have been validated by experiments.

Here, we presented a pipeline based on a series of deep learning (DL) models for AMPs design selectively targeting *C. acnes*, while guaranteeing non-hemolysis and novelty. Our training dataset is mostly constructed from the Database of Antimicrobial Activity and Structure of Peptides (DBAASP)^25^. To augment the data for targeted *C. acnes* inhibition, we constructed a phylogenetic tree to select bacteria species closely related to *C. acnes*. The deep learning models start with a basic generative model trained on all active AMPs sequences. Following that, the basic generative model was fine-tuned on the augmented *C. acnes*-specific data. Further, we trained two classifiers, one for predicting the antimicrobial activity and the other for predicting hemolysis. Then, we applied length filtering and clustering to prioritize the selections of peptides for in vitro experiments. At last, we synthesized 42 peptides to conduct in vitro tests and verified their antimicrobial potencies, along with no hemolysis and cytotoxicity.

In brief, our contributions are three-folds as follows:

- We proposed a data curation process, specially constructed a *C. acnes-*associated AMPs dataset by phylogenetic analysis.
- We designed a series of AI models including two generative models and two classification models to generate and classify unique and potent peptides in an efficient way.
- We selected 42 designed peptides to synthesize, and conducted in vitro tests to verify their antimicrobial potencies, hemolysis and cytotoxicity.

## Results

**Figure 1:**
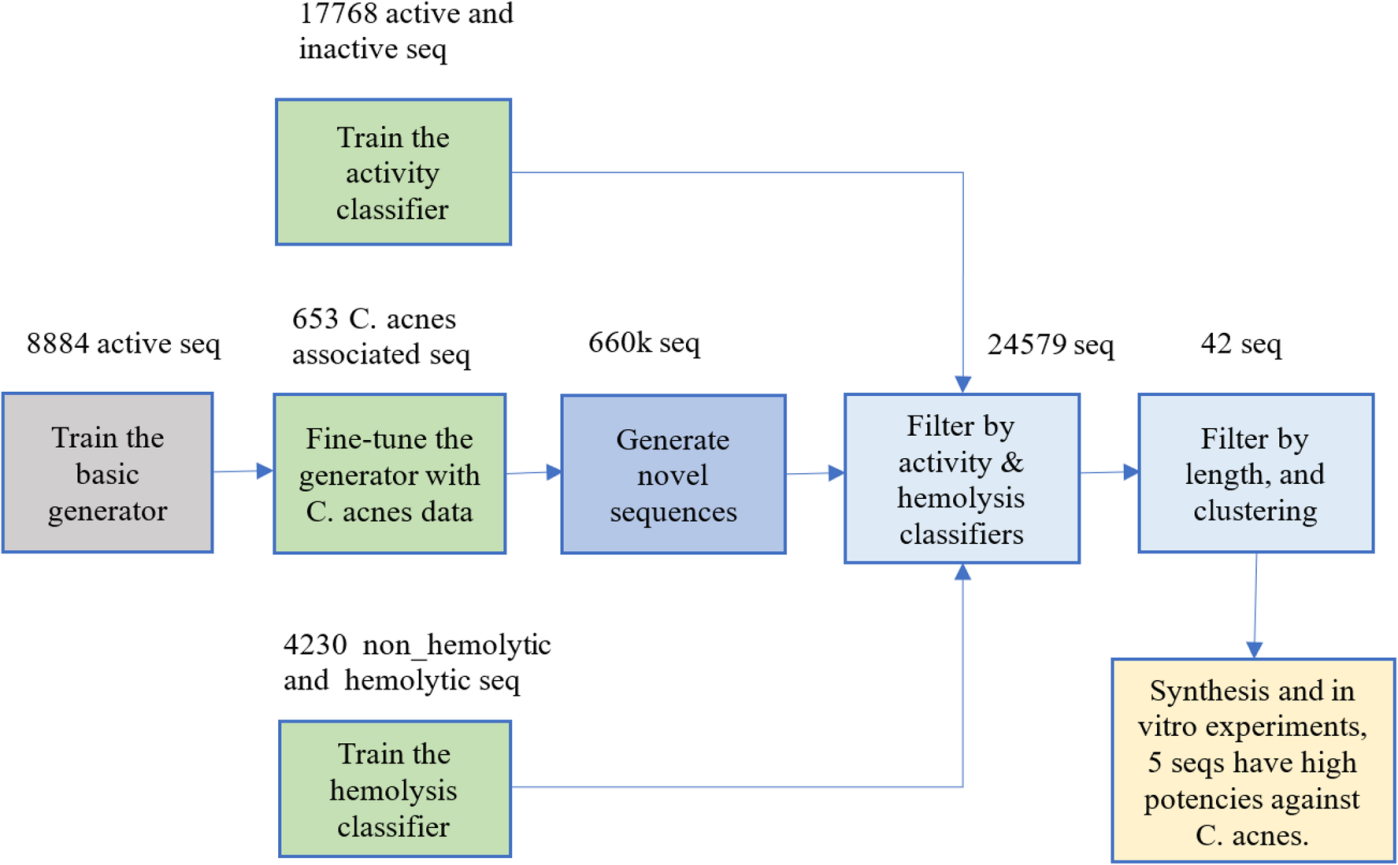
The pipeline overview.

Our design pipeline is shown in Fig. 1 and summarized as follows:

1. Basic generator training: we trained a basic initial DL generator with all known active AMPs sequences.
2. Fine-tuning for *C. acnes*: we fine-tuned the basic DL generator with *C. acnes*-related sequences, resulting in an anti-*C. acnes* generator model.
3. Activity and hemolysis classifiers training: we separately trained the activity and hemolysis DL classifiers.
4. Novel sequence generation: the anti-*C. acnes* generator was used to generate 660,000 novel sequences.
5. Classifier filter: the activity and hemolysis classifiers were used to filter the generated novel sequences, yielding 24,579 sequences.
6. Length filtering and clustering: we applied a length filter and sequence clustering algorithm to select 42 peptides for in vitro experiments.
7. In vitro validation: five of the 42 tested peptides demonstrated high selectivity and potency with minimum inhibitory concentration (MIC) values as low as 2–4 µg/mL against *C. acnes*.

### Datasets

We constructed our training set based on DBAASP^26^, which is a manually-curated database that contains over 19,000 peptides annotated with antimicrobial activity values and hemolytic values^25^. We selected peptides with lengths from 4 to 50 amino acid residues inclusive and with specified target species, leading to the creation of multiple datasets. The data curation methods are described in the Supplementary Materials.

#### Antimicrobial activity classification dataset

This dataset compasses 8884 active and 4009 inactive peptides from DBAASP, supplemented with 4875 additional pseudo-inactive sequences.

Hemolysis Classification Dataset. We curated 2217 hemolytic and 2013 non-hemolytic peptides from DBAASP.

#### Peptide generation dataset

To train the initial generator, we utilized all active AMPs sequences, irrespective of the target species. To fine-tune the initial generator with *C. acnes*-related AMPs, we constructed a phylogenetic tree and identified 28 species related to *C. acnes*.

**Figure 2:**
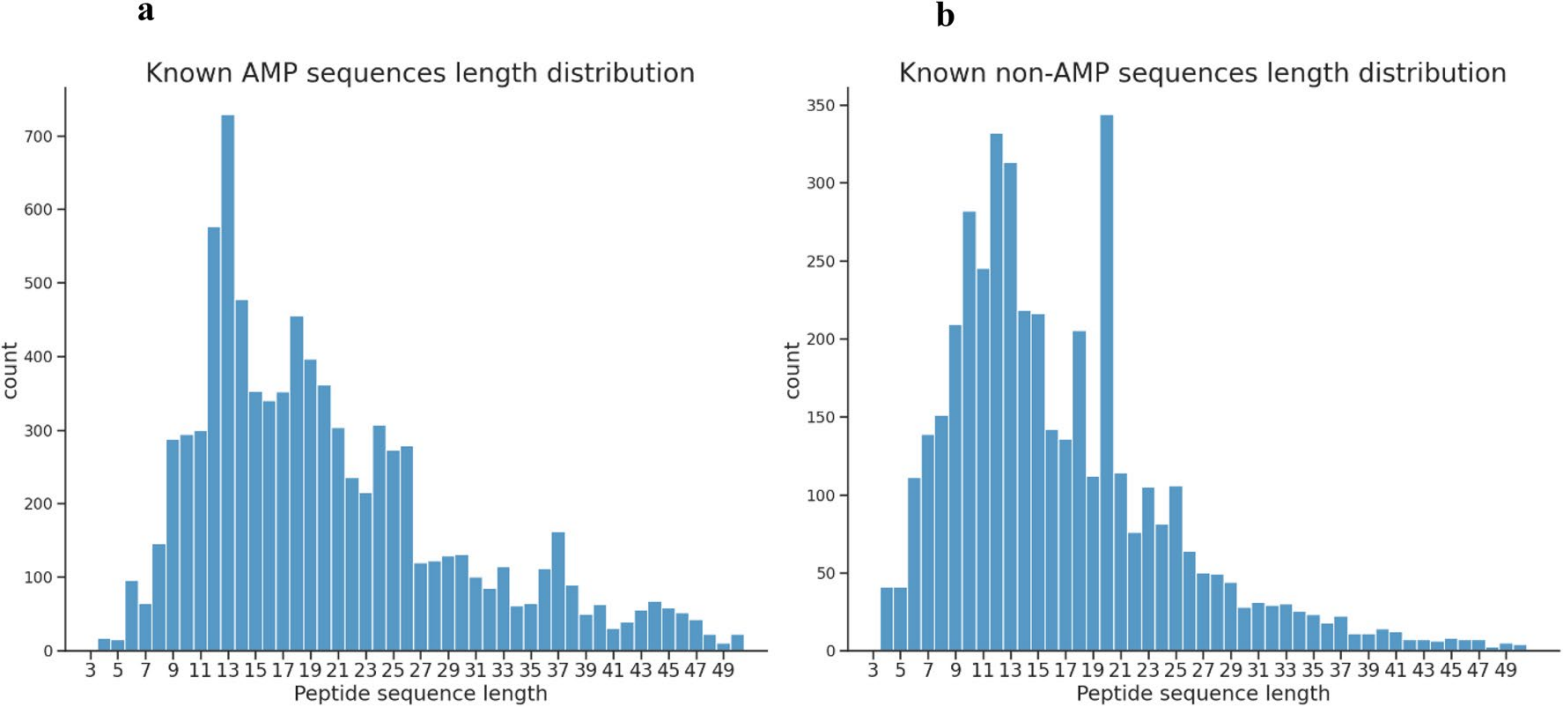
Sequences length distribution of (**a**) known active (AMP), and (**b**) known inactive (non-AMP).

Figure 2a,b show the length distribution of AMPs and non-AMPs. It is clear that most AMPs and non-AMPs gravitate towards the 9–21 amino acid span. Notably, the most prevalent lengths for AMPs peptides are 12, 13, and 14 amino acids. This prevalence might be attributed to cost constraints, given that over 80% of peptides in DBAASP are synthetically produced^26^.

### Generators

We first trained a basic generative model with recurrent neural network (RNN) networks with the known active peptides from DBAASP. Subsequently, we employed transfer learning techniques, fine-tuning this model using *C. acnes*-related sequences, resulting in the specialized anti-*C. acnes* AMPs generator (Fig. 1). This refined generator produced 660,000 sequences with a uniqueness ratio of 99.16% and a novelty ratio of 99.97%. Here, the novelty is defined as the absence of a sequence in the curated known active and inactive training sequences in DBAASP, to check the generator performance.

Comparing key physicochemical attributes of both known and generated AMPs offers insights into our generative model’s efficacy. In the comparisons below, we defined three groups of data: “Known general AMPs” representing the 8884 known active AMPs in DBAASP with documented antimicrobial activity against at least one bacterial species; “Known anti-*C. acnes* AMPs”, a subset of “Known general AMPs”, representing the 653 active and non-hemolytic AMPs against *C. acnes* or related species; “Generated anti-*C. acnes* AMPs” representing the 660,000 sequences generated in this study.

**Figure 3:**
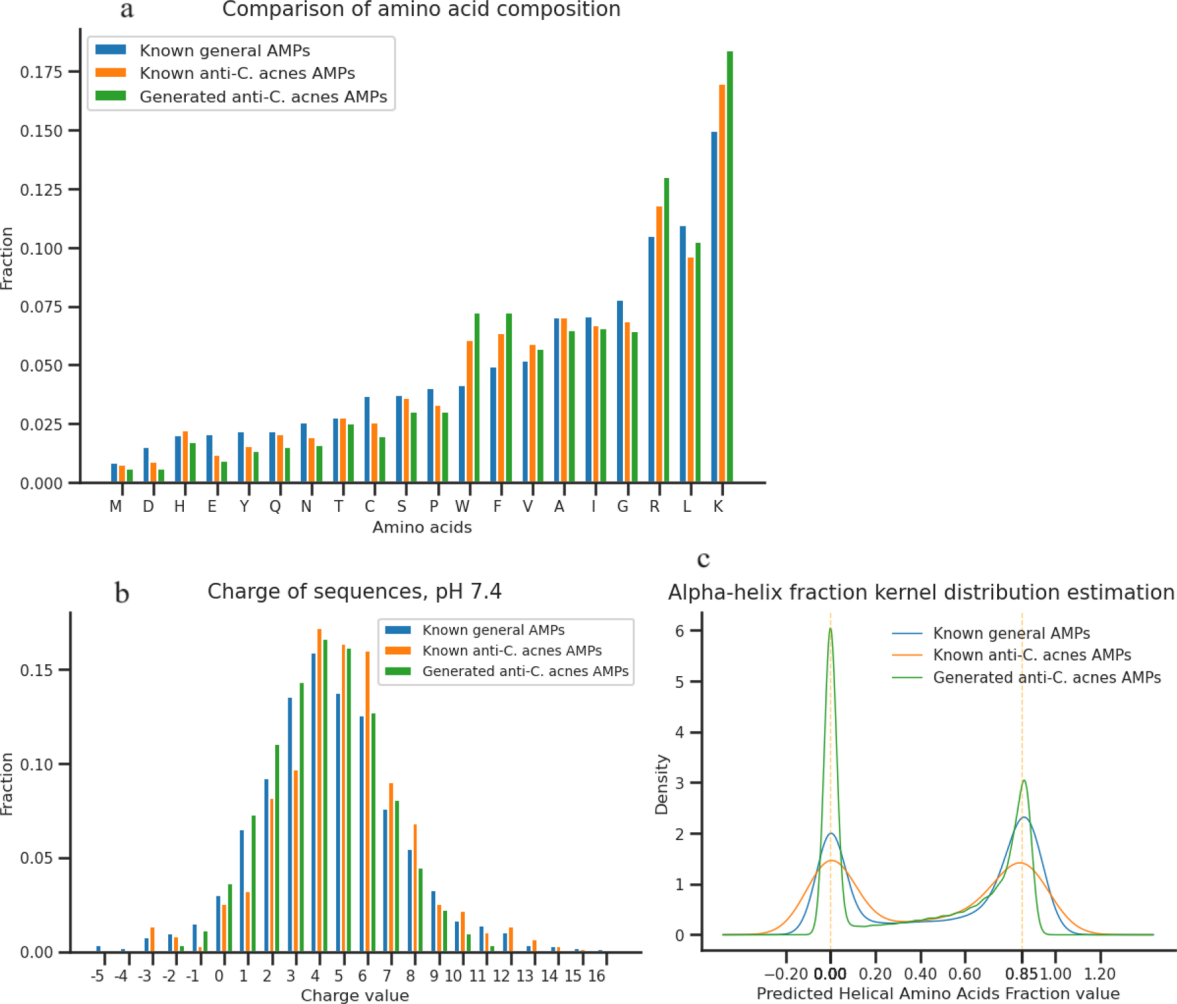
Comparison of physicochemical properties. (**a**) comparison of amino acid composition, (**b**) charge of sequences at pH 7.4, (**c**) predicted alpha-helical amino acids fraction.

Visual inspection of amino acid composition (AAC) distributions (Fig. 3a) indicates a closer resemblance between “Generated anti-*C. acnes* AMPs” and “Known anti-*C. acnes* AMPs” compared to “Known general AMPs”. Statistical testing also supports such observations. At the significance level of 0.05, the differences of each amino acid proportions show that for all amino acids, the distributions between “Generated anti-*C*. *acnes* AMPs” and “Known anti-*C*. *acnes* AMPs” do not significantly differ, whereas 13 out of 20 amino acids show significant differences when comparing “Generated anti-*C*. *acnes* AMPs” to “Known general AMPs”. The AAC statistical analysis data are in Supplementary Table 2. These results validate the closer AAC resemblance between the generated and known anti-*C*. *acnes* AMPs and demonstrate our transfer learning strategy’s effectiveness.

Although charge distribution across the datasets was largely consistent, distinct variations appeared at specific charge levels (Fig. 3b). Kernel distribution estimations (KDE) for anticipated α-helix amino acids fraction^27^ showcased consistent dual peaks at the value 0.00 and 0.85 across the datasets (Fig. 3c). This pattern suggests that the generated anti-*C. acnes* AMPs predominantly feature positive charges and are inclined to assume an amphipathic α-helix structure, aligning more with known anti-*C. acnes* than known general AMPs. The Boman index^28^ and hydrophobic moment^29^ values distributions for generated anti-*C. acnes* AMPs aligned closely with both known anti-*C. acnes* and known general AMPs (Supplementary Fig. 1a,b). Such observations spotlight the distinct physicochemical profiles across the known general, known anti-*C. acnes*, and generated anti-*C. acnes* AMPs, offering crucial insights for targeted therapeutic implementations. We used modlAMP^30^ to calculate the physicochemical attributes of peptides.

### Classifiers

**Figure 4:**
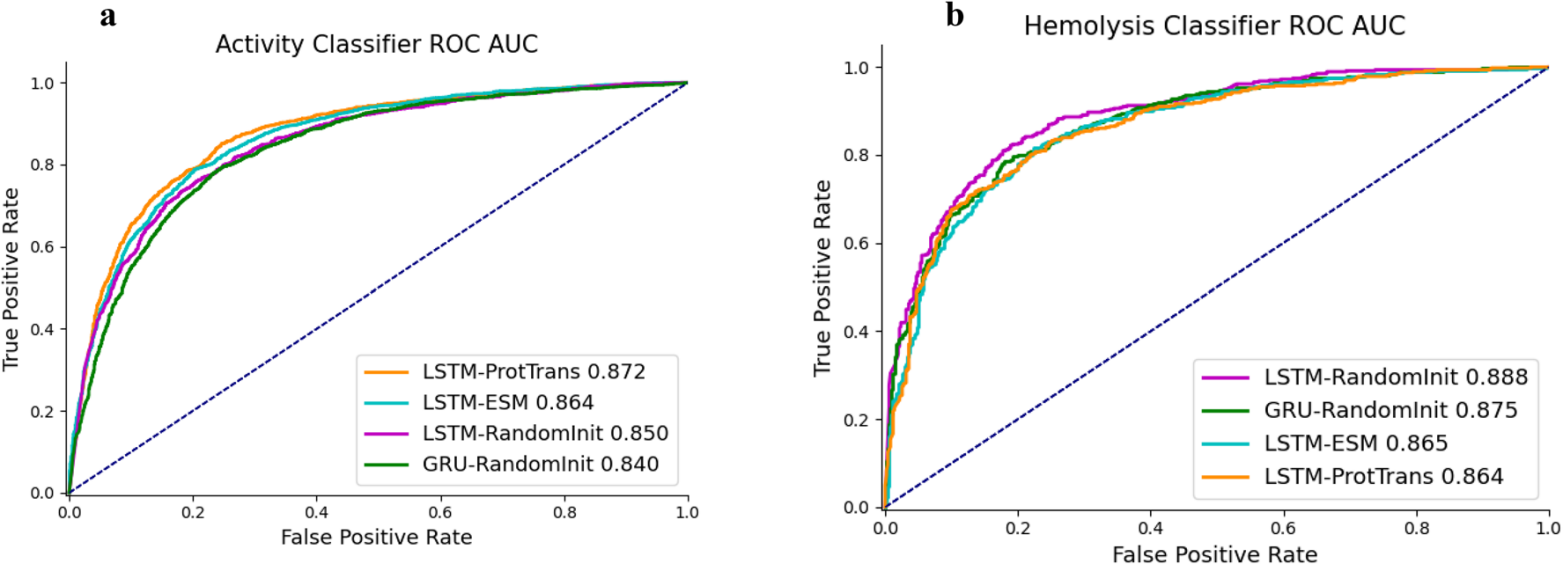
Classifiers’ performances. (**a**) The activity classifier ROC AUC, (**b**) the hemolysis classifier ROC AUC. The probabilistic prediction values were converted into binary classification values using a threshold of 0.5.

In our assessment of gated recurrent unit (GRU)^31^ and long short-term memory (LSTM)^32^ architectures, we also benchmarked various pre-trained protein language model embeddings. The experimental outcomes are illustrated in Fig. 4a,b. Comparing GRU and LSTM using random initial embeddings, LSTM marginally outperformed GRU. Subsequently, we compared random initial embeddings against two popular pre-trained protein language model embeddings: Evolutionary Scale Modeling (ESM)^33^ and ProtTrans^34^, deploying them with LSTM. Among the configurations tested, LSTM paired with ProtTrans performed best for the activity classification across all computed metrics (ROC AUC = 0.872, AUPRC = 0.854, accuracy = 0.792, precision = 0.785, recall = 0.803, F1 score = 0.794). As shown in Fig. 4a, ROC AUC was used as comparison metrics for different classifiers. In contrast, for hemolysis classification, LSTM using random initial embeddings outshone other configurations across most computed metrics (ROC AUC = 0.888, AUPRC = 0.879, accuracy = 0.814, precision = 0.807, recall = 0.805, F1 score = 0.806), shown in Fig. 4b. Interestingly, activity classifiers exhibited subdued performance relative to hemolysis classifiers, insinuating a higher sequence diversity and intricacy in activity than in hemolysis.

In the larger activity dataset of 17,768 sequences, both ESM and ProtTrans pre-trained protein embeddings boosted the classifier performances by roughly 2%. Conversely, for the hemolysis dataset, encompassing 4230 sequences, these pre-trained embeddings decreased performance. We speculate that pre-trained protein, predominantly trained on longer protein sequences, may not consistently excel with AMPs datasets. This is attributed to AMPs sequences often being shorter, spanning from 4 to 50 amino acids, which is a stark contrast to the typical protein sequences. Such insights suggest that leveraging pre-trained protein embeddings in AMPs models might hinge on the specific AMPs dataset’s size and characteristics.

To ensure a minimized false positive rate, we set threshold values above 0.99 to transform the final two classifiers’ probabilistic outputs to binary classifications. Consequently, the activity and hemolysis classifiers demonstrated precisions of 0.906 and 0.917, respectively. Sequentially applying both yielded a collective precision of 0.828, filtering down to 24,579 sequences.

We emphasized that the goal of this paper is not to provide AMP prediction models that outperform existing ones. Rather, the goal is to build a pipeline with comparable accuracies and selection strategies to design novel and potent peptides anti specific strains, such as *C. acnes*. We believe the performances of our models are sufficient for the intended task. They are also able to identify potential AMPs that might be overlooked by conventional methods (as detailed in Table 1 of the Supplementary Materials).

### Length and novelty filters

We chose peptide sequences with lengths ranging from 10 to 15. To guarantee the novelty of these peptides, they were mandated to have a minimum of five mutations distinct from known AMPs in the DBAASP database, avoiding trivial analogs of known AMPs. For added diversity, we clustered the peptides based on their Levenshtein sequence distances. This rigorous process yielded 42 peptides for further in vitro experiments, and their sequences and physicochemical properties are in Supplementary Table 3. This length and novelty filter flowchart is shown in Supplementary Fig. 2.

### In vitro experiments

#### In vitro antimicrobial activity of designed peptides

We synthesized a set of 42 peptides and evaluated in vitro inhibitory activity against *C. acnes* at concentrations of 100/50/25/12.5 µg/mL. A peptide was deemed to have antimicrobial activity if *C. acnes* growth was inhibited by more than 50% at a given concentration. The results yielded 14 high activity, 16 medium activity, 4 low activity, and 8 non activity peptides (Table 1). Remarkably, our design’s success rate for effective antimicrobial peptides was 71.4% (considering only medium and high activity peptides), showcasing exceptional efficacy.

**Table 1:** Summary and classification of antimicrobial activity of tested peptides against *C. acnes*.

Category	Concentration with antimicrobial activity > 50% (µg/mL)				Peptides ID
	100	50	25	12.5	
High activity	Inhibit	Inhibit	Inhibit	Inhibit	AMP-5,8,9,10,11,12, 14,21,25,27,29,31,33,38
Medium activity	Inhibit	Inhibit	Inhibit	No	AMP-2,13,15,16,17, 18,19,20,23,39,41
Medium activity	Inhibit	Inhibit	No	No	AMP-1,4,7,22,32
Low activity	Inhibit	No	No	No	AMP-3,6,26,30
No activity	No	No	No	No	AMP-24,28,34,35, 36,37,40,42

**Figure 5:**
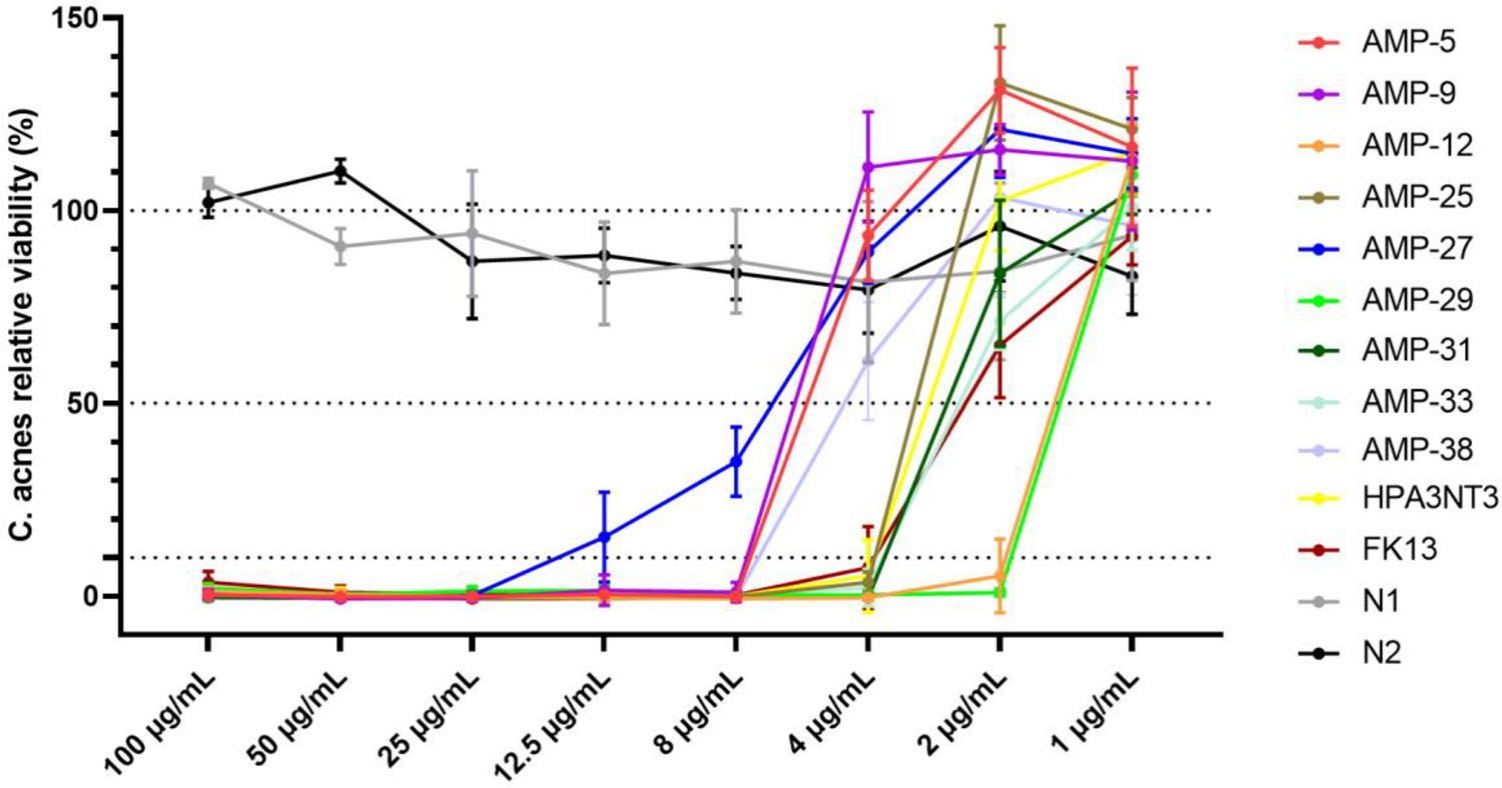
*C. acnes* viability with peptides at different concentrations. MIC of AMP-29,12, 25, 31, 33 are 2–4 µg/mL.

To pinpoint the MIC of the most potent AMPs, we assessed their inhibitory effects against *C. acnes* at even lower concentrations: 8/4/2/1 µg/mL. As depicted in Fig. 5, AMP-29,12 displayed the lowest MIC, registering at just 2 µg/mL. This was closely followed by AMP-25,31,33 and the positive controls (HPA3NT3 and FK13), all recording MICs of 4 µg/mL. AMP-5,9,38 marked their MICs at 8 µg/mL (Table 2). These impressively low MICs highlight the robust inhibitory prowess of AMPs against *C. acnes*.

**Table 2:** MIC and toxicity of tested peptides. MIC is determined as the lowest test concentration of peptides for microbial viability less than 10%. EC90 is the highest test concentration of peptides for rabbit red blood cell or HaCaT cell viability higher than 90%. Index is the ratio of EC90 to MIC of *C. acnes*.

	MIC (µg/mL)				Cytotoxic		Hemolysis	
	*C. acnes*	*E. coli*	*S. aureus*	*C. albicans*	EC90 (µg/mL)	Index	EC90 (µg/mL)	Index
AMP-5	8	12.5	100	12.5	80	10	240	30
AMP-9	8	25	> 100	25	160	20	240	30
AMP-12	2	8	8	25	32	16	80	40
AMP-25	4	50	> 100	25	80	20	240	60
AMP-27	25	100	> 100	50	> 320	12.8	160	6.4
AMP-29	2	8	8	25	32	16	64	32
AMP-31	4	8	8	25	32	8	80	20
AMP-33	4	25	50	12.5	80	20	80	20
AMP-38	8	8	12.5	50	80	10	128	16
HPA3NT3	4	50	100	25	32	8	128	32
FK13	4	8	25	–	80	20	64	16

**Figure 6:**
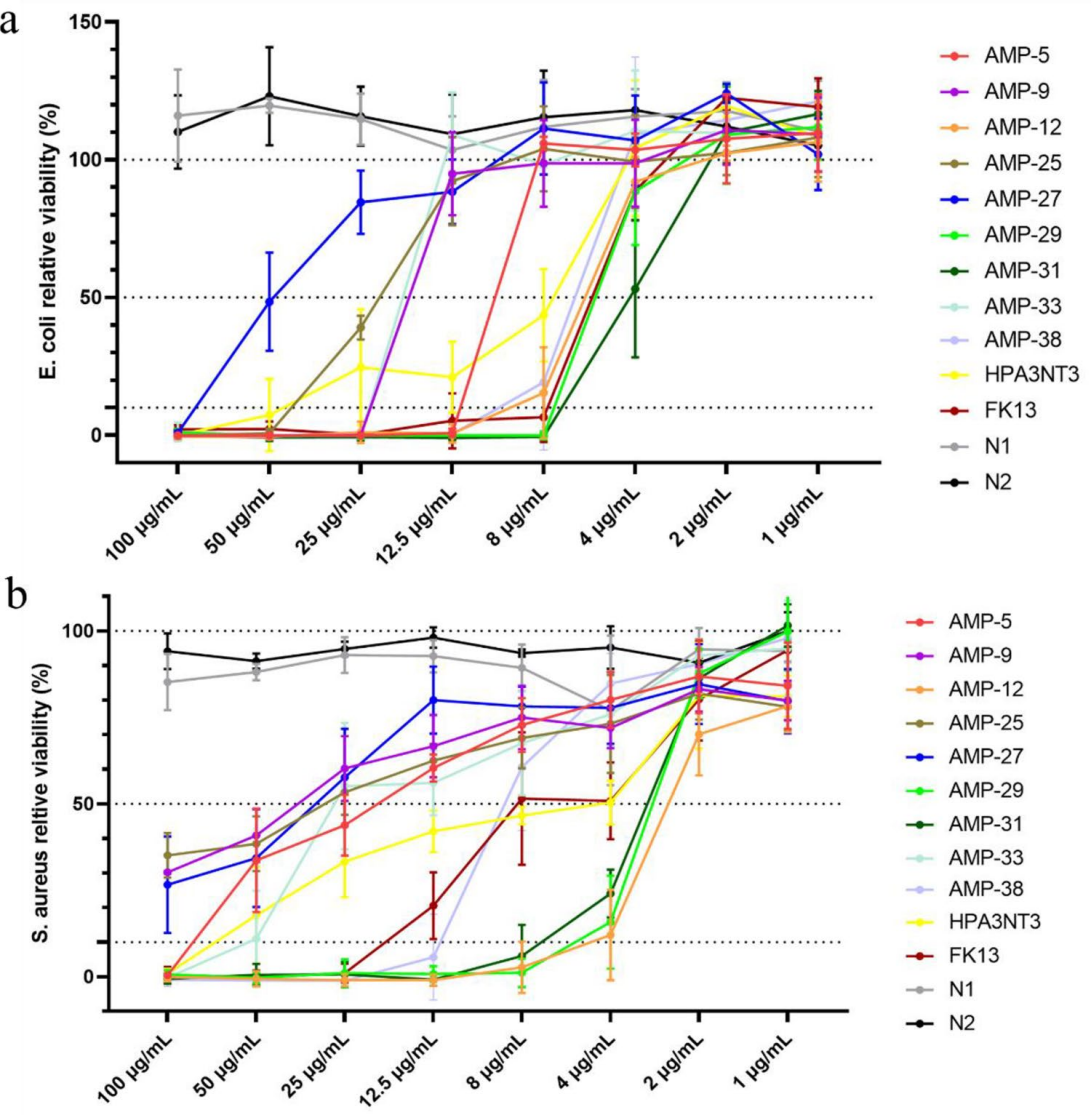
Viability of *E. coli* (**a**) and *S. aureus* (**b**) with peptides at different concentrations.

To ascertain the broad-spectrum efficacy of these peptides, we evaluated their activity against *E. coli* and *S. aureus*, representative of Gram-negative and Gram-positive bacteria, respectively. As illustrated in Fig. 6, every peptide we assessed effectively inhibited both *E. coli* and *S. aureus*, confirming their broad-spectrum antimicrobial activity, in line with previously studied AMPs^7,36–38^. It’s noteworthy that the MIC values for *E. coli* and *S. aureus* were higher than those for *C. acnes* (Table 2). This aligns with our design, as these peptides were specifically engineered to target *C. acnes*.

**Figure 7:**
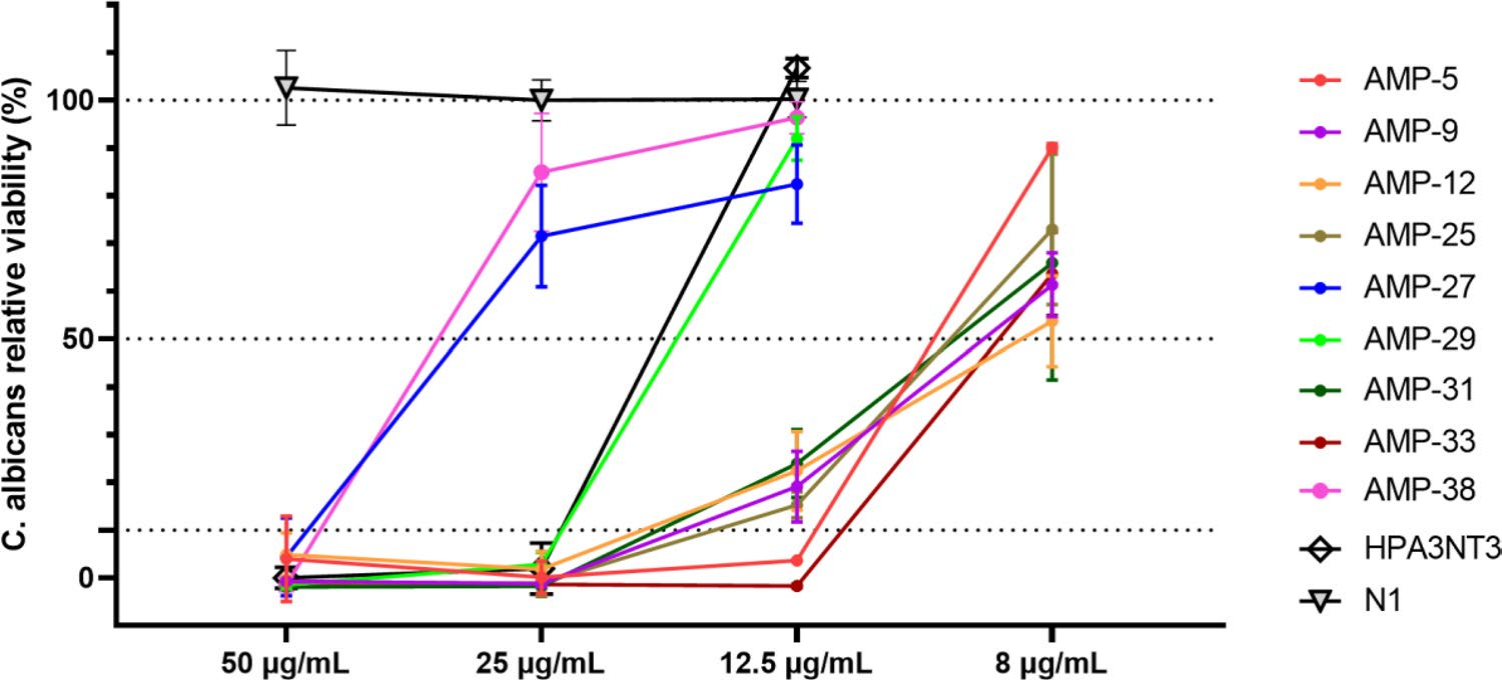
*C. albicans* viability with peptides at different concentrations.

Furthermore, we assessed the antimicrobial efficacy of the AMPs against *C. albicans*, a typical fungus. As demonstrated in Fig. 7, all evaluated peptides effectively inhibited *C. albicans*, indicating their capability to combat both bacterial and fungal pathogens. However, it’s worth noting that the MIC values for *C. albicans* were also higher than those for *C. acnes* as detailed in Table 2. In essence, these comprehensive antimicrobial tests underscore the potent and broad-spectrum activity of our designed AMPs.

#### Hemolytic activities of designed peptides

**Figure 8:**
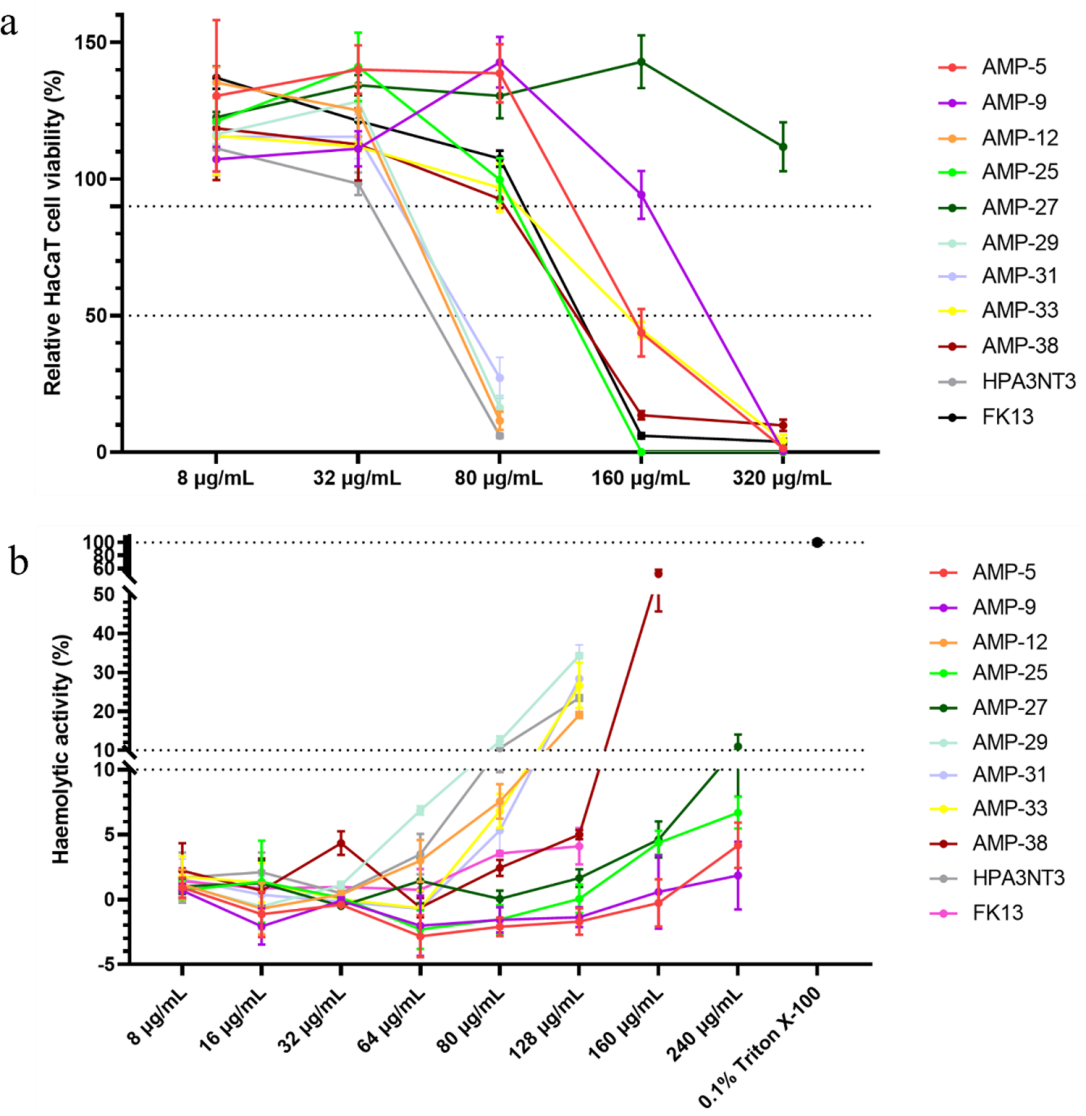
Toxicity of peptides. (**a**) Hemolysis of erythrocytes (2% v/v) with peptides at different concentrations, (**b**) cell viability of HaCaT with peptides at different concentrations.

The hemolytic activity, a pivotal metric in therapeutic AMPs development, gauges potential toxicity to erythrocytes. Figure 8a shows the hemolytic behavior of the examined peptides relative to the 0.01% Triton X-100 control, which yields a 100% erythrocyte lysis. It’s demonstrated that the hemolytic concentrations (EC90) of all tested peptides are significantly higher than their respective MICs against *C. acnes*, which means these peptides are very safe. Among them, AMP-5,9,25 showcased the greatest erythrocyte tolerance, tolerating concentrations exceeding 240 µg/mL. This was followed closely by AMP-27,38, and HPA3NT3. However, the most potent antimicrobial peptides, namely AMP-12,29,31,33, and FK13, exhibited lower tolerance, possibly due to their distinct structural properties. Overall, these findings affirm that our custom-designed AMPs present minimal erythrocyte toxicity.

#### In vitro cytotoxic activities of designed peptides

For anti-acne applications, it's vital to assess peptide cytotoxicity on human skin cells. Hence, we evaluated our designed peptides against human keratinocytes (HaCaT) using the CCK-8 assay. Untreated cells served as the baseline, representing 100% viability. As depicted in Fig. 8b, at lower concentrations, peptides tended to enhance HaCaT cell viability. However, at elevated concentrations, they inhibited cellular activity. Notably, AMP-27 stood out, demonstrating the highest cell tolerance up to 320 µg/mL. This was followed by AMP-9 at 160 µg/mL and a cohort of peptides, including AMP-5,14,21,25,33,38, all showing tolerance up to 80 µg/mL. When combined all the data from cytotoxic, antimicrobial, and hemolytic activities (refer to Table 2), a pattern seems to emerge: peptides with heightened antimicrobial prowess often exhibited increased cytotoxicity.

## Conclusion

In this study, we introduce a sophisticated pipeline tailored for the design of AMPs, specifically targeting *C. acnes*, while ensuring their novelty and minimizing hemolytic activity. Our primary data, consisting of both active and inactive peptides as well as hemolytic and non-hemolytic variants, was derived from DBAASP. Through a constructed phylogenetic tree, we pinpointed species closely related to *C. acnes*, which allowed us to curate a dataset of *C. acnes*-associated AMPs. Leveraging this dataset, we crafted models for AMPs generation, activity classification, and hemolysis prediction. Notably, our exploration into pretrained protein embeddings revealed differential performance: while ProtTrans excelled in large activity datasets, random initial embeddings shined in smaller hemolysis datasets.

From our *C. acnes*-focused generator, we extracted 660,000 sequences. Subsequent stringent filtration through classifiers, along with considerations of peptide length and novelty, culminated in 42 shortlisted peptides primed for synthesis, predominantly 14–15 residues long. Laboratory validations were overwhelmingly positive: 30 unique peptides exhibited inhibition against *C. acnes* growth, yielding a 71% success rate. Standout peptides like AMP-12/29/31/33 showcasing incredibly low MIC values of 2/4 µg/mL, yielding a 10% success rate. Impressively, several designed AMPs, including AMP-5/9/12/25/27/29/31/33/38, also inhibited *E. coli*, *S. aureus*, and *C. albicans*, albeit at higher MICs. This underlines the broad-spectrum yet selective antimicrobial prowess of our designed AMPs. Further bolstering their therapeutic potential, all evaluated AMPs showcased minimal hemolytic and cytotoxic activities. As *C. acnes* are the most important causes of acne vulgaris which affect more than 80% of all adolescents and young adults worldwide, these AMPs could provide promising anti-*C. acnes* therapies in both pharmaceutical and cosmetic fields.

The selectivity of the designed AMPs, as observed, aligns well with our design intention: showcasing robust inhibitory activity against *C. acnes* while presenting diminished effects on other microbes. We think the underpinning mechanism behind this selectivity is mostly caused by the transfer learning with *C. acnes*-centric AMPs sequences.

In summary, the AI-driven pipeline presented here is efficient and scalable, and can be tuned to not only for anti-*C. acnes* peptides, but also for generating and filtering other unique anti-microbial functional peptides for diverse applications from healthcare to cosmetics.

## Methods

### Generators

The methodology employed hinges on the power of deep learning, specifically using the PyTorch^39^. The peptide sequences were broken down into individual amino acids for tokenization, with each amino acid represented by a unique identifier. This method of representation transforms biological sequences into a computationally manageable format.

For training, the dataset, which is made up of 8884 active AMPs, was divided using the standard 75% training and 25% testing split. This basic generator was subjected to 300 training epochs, guided by the cross-entropy loss function. The weight adjustments during training were managed by the Stochastic Gradient Descent (SGD)^40^ optimizer, with momentum incorporated to enhance the convergence speed, with a momentum of 0.9 and a learning rate of 0.005. When it came to generating new sequences, the process started with an initiating ‘start’ token. The subsequent tokens were iteratively generated based on the hidden state of the previously generated token, a procedure that continued until an ‘end’ or a ‘padding’ token was produced. Interestingly, while both the GRU and LSTM architectures were tested, they delivered almost identical performances for the generative task when dealing with short peptide datasets. Given that GRUs offer a computational speed advantage over LSTMs, the choice was made to utilize GRUs for the generative model. This model is structured with an embedding layer (for token-to-weight matrix transformations), a GRU layer (for temporal feature extraction), a dropout layer (to deter overfitting), and a dense output layer for the prediction of probabilities. The entire architecture is illustrated in Supplementary Fig. 3a.

For fine-tuning toward *C. acnes*-specific AMPs, a subset of 653 *C. acnes*-related AMPs was used. The fine-tuning process lasted 300 epochs and was optimized using SGD. It’s noteworthy that the learning rate used for fine-tuning was a mere tenth of what was used for the initial basic generator training. This transfer learning approach was harnessed to capitalize on the foundational learnings of the basic generator, subsequently refining it for the specific task of anti-*C. acnes* AMPs design.

### Classifiers

The classifiers for both activity and hemolysis utilized the same model architecture. This included an embedding layer to transform the input sequence of token IDs (each representing an amino acid) into a dense representation. This was followed by an LSTM layer, which is well-suited to handle sequential data, to extract and recognize patterns within the peptide sequences. A dropout layer was incorporated next as a regularization method to reduce overfitting, by randomly setting a fraction of input units to 0 at each update during training time. Following this, a hidden dense layer was introduced, followed by a ReLU^41^ activation layer, which helped in introducing non-linearity to the model. The architecture concluded with a dense layer that outputted the predicted probabilities. Within the context of the hemolysis classifier, non-hemolytic sequences were labeled as positive while hemolytic sequences were labeled negative. The entire architecture is illustrated in Supplementary Fig. 3b.

From the ProtTrans suite, we chose the ProtT5-XL-UniRef50 model, notable for its expansive embedding size of 1024. From the ESM suite, our choice was the esm2_t33_650M_UR50D model, which offers an even larger embedding dimensionality of 1280. To ensure a level playing field during comparisons, we fixed the embedding size of our random initial embeddings at 1280, which matches the highest dimensionality of our selected pre-trained embeddings.

Our empirical evaluations, as depicted in Fig. 4, illuminated our decision to adopt the LSTM architecture in tandem with the ProtTrans pre-trained protein embeddings for our activity classifier. In contrast, for the hemolysis classifier, the LSTM model combined with the randomly initialized embeddings demonstrated superior performance.

The training regimen for the classifiers was consistent across both activity and hemolysis tasks. We employed a training duration of 150 epochs, guided by the cross-entropy loss function. Optimization was achieved via SGD equipped with a momentum parameter set at 0.9 and a learning rate initialized at 0.01. To enhance the learning process, we implemented the cosine annealing learning rate schedule.

### Length and novelty filters

In our pursuit of optimal AMPs, several guiding principles informed our selection of peptide lengths. Initially, our focus on shorter peptides was driven by practical considerations. Shorter sequences are less costly to produce, a critical factor when contemplating their future commercial viability.

Delving deeper, our analysis of the DBAASP database offered further insights. It revealed that the predominant lengths for effective AMPs were 12, 13, and 14 amino acids. Remarkably, there was a stark paucity of active AMPs shorter than 9 amino acids as shown in Fig. 2. With these findings, our choice to encompass peptide lengths ranging from 10 to 15 was strategic, aiming to encompass the bulk of active peptides while also expanding the scope slightly to capture any overlooked potential.

A significant consideration was the potential structure these peptides might adopt. We theorized that extremely truncated peptides might struggle to form the alpha-helical structures typical of many AMPs. Such structures are pivotal for AMPs functionality. As a result, we delineated 10 amino acids as the minimal peptide length, ensuring each peptide maintained the structural integrity vital for efficacy.

Variability and uniqueness in AMPs were other aspects we were keen to emphasize. To uphold this diversity, we applied clustering based on sequence Levenshtein distances, establishing a threshold distance of 8 for this purpose. From each formed cluster, we chose its center. Moreover, for those clusters teeming with over 10 sequences, an extra sequence was arbitrarily picked. The calculating Levenshtein distances was handled by RapidFuzz 3.3.0. For clustering, we used RDKit 2023.09.2.

### Peptide synthesis

The peptides were synthesized by GL Biochem Ltd. (Shanghai, China) using Fmoc solid phase technology. The purity of the peptides determined by high-performance liquid chromatography (HPLC) was > 95%. HPA3NT3 and FK13 are used as positive control peptides, and N1/N2 are used as negative control peptides^35,42^. All peptides were soluble in water or Phosphate buffered saline (PBS). In total, 44 peptides are synthesized and conducted in vitro tests, and their sequences are given in Supplementary Table 4.

### Microbial preparation and culture

Strains of *E. coli* (ATCC 25922) and *S. aureus* (ATCC 25913) were grown in Luria Bertani (LB, Sangon Biotech, A507002) medium at 37 °C at 180 rpm under aerobic conditions.

The strain of *C. acnes* (BNCC 336649) was cultured in Wilkins-Chalgren Anaerobe Broth (QingDao RiShui Bio-technologies, 11071) at 37 °C under an anaerobic atmosphere performed by a Labiophy HL-B automatic hypoxic workstation (Dalian, Liaoning Province, Labiophy HL-B), inflated with a gas mix of 15% carbon dioxide, 83% nitrogen, and 2% hydrogen.

Strains of *C. albicans* (ATCC 14053) were grown in Nodule Bacteria Medium YM broth (Beijing Solarbio Science and Technology, LA6970) at 28 °C at 180 rpm under aerobic conditions.

Logarithmic phase growth bacteria were used for antimicrobial testing.

### In vitro experiments

#### In vitro antimicrobial testing

To determine the MIC values, the broth microdilution method was used. MIC values of peptides were determined as the lowest concentrations of test samples that inhibit 90% bacterial growth, which was measured by optical density^7^. Briefly, peptide samples were prepared as a stock solution in water and then diluted. 180 μL of the bacterial suspension (10^7^ CFU/mL) and 20 μL of the tested sample were added together into 96-Well Deep Well Plates and incubated 24 h for *E. coli* and *S. aureus* or 48 h for *C. acnes* and *C. albicans*. For each test, two columns of the plate were kept for sterility control (broth only) and growth control (broth with bacterial inoculums, no antibiotics). The optical density of cultures was measured at 630 nm by a microplate reader (SYNERGY-H1, BioTek, USA) to estimate bacterial growth. All experiments were conducted in triplicate. We used ciprofloxacin (CPFX) as the antibiotic control for antimicrobial testing.

#### Hemolytic activity assay

The hemolytic activity of peptides was assayed on rabbit red blood cells (rRBCs)^43^. Briefly, 2% rRBCs (150 μL) were incubated in the presence of various peptide concentrations (50 μL) at 37 °C for 2 h. After centrifugation, the supernatants were collected for measuring absorbance at 545 nm. PBS and 0.01% Triton X-100 were used as the negative (no hemolysis) and positive (100% hemolysis) control, respectively. Triplicate tests were performed.

#### Cell culture and viability assay

HaCaT (IM-H225) cells from Xiamen Immocell Biotechnology Co., Ltd were cultured in DMEM high glucose medium (4.5 g/L, Gibco, USA), supplemented with 10% fetal bovine serum (Gibco, USA), 100 U/mL of penicillin and 100 μg/mL of streptomycin in a 5% CO_2_ fully humidified environment at 37 °C.

Cell Counting Kit-8 (CCK-8; Biosharp, China) was used to assess the rate of cellular proliferation and quantify cell viability^44^. In brief, HaCaT cells were seeded in 96-well plates with 100 μL of medium at a density of 1 × 10^4^ cells per well. After incubation of cells overnight, peptides were added into the medium for another 24 h incubation. Then, 10 μL of CCK8 solution was applied to each well and incubated for 1 h at 37 °C. Finally, the absorbance values at 450 nm were determined using a microplate reader (SYNERGY-H1, BioTek, USA). Two columns of the plate were kept for negative control (medium only) and growth control (medium with HaCaT cell). All experiments were conducted in triplicate.

### Supplementary Information

Supplementary Table 1.

Supplementary Table 2.

Supplementary Table 3.

Supplementary Table 4.

Supplementary Table 5.

Supplementary Information.

## Data Availability

The datasets generated during the current study and supplementary materials are available in the Zenodo repository, https://zenodo.org/records/10471900?token=eyJhbGciOiJIUzUxMiJ9.eyJpZCI6ImY5NGJlMDFkLTlhYjItNGZhYS05MTAyLTYzNGNlZDUzMDY3MCIsImRhdGEiOnt9LCJyYW5kb20iOiI2MDA3NDc0MWZlM2YyZTgzY2MzYTk0YTEzODZiYmIwYyJ9.Bt2UHetiACr-D4ixlNyAORBllFu93MVSieoDuu8WkBvTkbOSWtBJBvZJjbDKR-wEUCF0hyoYYMVNhVDeEXcN-A.
