# Supplementary material for: Novel antimicrobial peptides against Cutibacterium acnes designed by deep learning
Source: Sci Rep. 2024 Feb 24;14:4529. doi: 10.1038/s41598-024-55205-3 (PMC10894229; doi:10.1038/s41598-024-55205-3)
Supplement: Supplementary file 6 — Supplementary Information. [file 41598_2024_55205_MOESM6_ESM.docx]

Supplementary Materials

Novel antimicrobial peptides against *Cutibacterium acnes* designed by deep learning

Qichang Dong^1,4^, Shaohua Wang^1,4^, Ying Miao^3,4^, Heng Luo^1^, Zuquan Weng^3^ and Lun Yu^2, *^

^1^ Shanghai MetaNovas Biotech Co., Ltd, Shanghai, 200120, China

^2^ Metanovas Biotech Inc., Foster City, 94404, USA

^3^ College of Biological Science and Engineering, Fuzhou University, Fuzhou, 350108, China

^4^ These authors contributed equally to this work: Qichang Dong, Shaohua Wang and Ying Miao.

* Correspondence: [lunyu@metanovas.com](mailto:lunyu@metanovas.com)

## Table of Contents

Methods of data curation

Comparison with Conventional Methods

Supplementary Figures 1 to 3

Supplementary Tables in separate spreadsheet files

Supplementary References

## Methods of data curation

### DBAASP

From the comprehensive AMP dataset procured from the DBAASP website^1^, we amassed a collection of 19,316 peptides. To ensure relevance and specificity, we implemented stringent criteria: the peptides needed to have a minimum length of 4, end with a free or amidated C-terminus or begin with an acetylated N-terminus, and exclusively comprise natural L-amino acids or their D-enantiomers.

Our classification system for activity relied on a dual-threshold metric. We labeled sequences as 'active' when they exhibited an activity concentration below 32 µg/ml or 10 µM and had at least one designated target species. In contrast, sequences that uniformly displayed activity concentrations surpassing 32 µg/mL or 10 µM were categorized as 'inactive' ^2^.

To discern between hemolytic and non-hemolytic activities, we standardized all recorded concentrations to µM, utilizing the metrics against human erythrocyte cells. We deemed sequences as 'non-hemolytic' if they demonstrated hemolytic values under 20% at concentrations of 50 µM or more. On the other hand, sequences presenting a hemolytic value equal to or exceeding 20% at any concentration were labeled as 'hemolytic'^2^.

Our final tally comprised 8,884 active peptides and 4,009 inactive peptides, along with 2,217 hemolytic peptides and 2,013 non-hemolytic peptides. It's crucial to note that the hemolysis dataset is inherently a subset of the broader activity dataset.

To foster a balanced dataset for our activity classifier, we embarked on the creation of 4,875 supplementary pseudo-inactive sequences. This process was bifurcated into two distinct strategies:

- Scrambling the sequences of active peptides, yielding 2,437 sequences. This involved rearranging the order of amino acids to derive sequences distinct from the parent active peptide, ensuring that the structural and functional attributes were disrupted. The step-by-step process is as below:

Step 1, randomly samples 2,437 sequences from 8,884 known active AMPs.

Step 2, splits each active sequence to 1 or 2 amino acid residue parts, and randomly adjusts the order of these parts and concatenate to create a new sequence with the same sequence length.

Step 3, if the "new" sequence is in available sequences which include both known active and inactive sequences, repeats step 2 to create a new sequence.

Step 4, if after a loop limit (i.e., 100) between step 2 and step 3, a new sequence cannot be got, returns a sequence by adding a random amino acid into the head or tail of the current scrambled sequence and stop this loop limit. The loop limit between step 2 and step 3 rarely triggers, potentially never. Then, gets 2,437 scrambled sequences.

Step 5, in most cases, any scrambled sequences are not in the available sequences. If some scrambled sequences are in the available sequences, deletes them, and repeats from step 1 with the deleted number as the new sampling number. Finally gets 2,437 scrambled novel sequences from active AMPs' sequences.

- Fragmenting sequences from the renowned SwissProt database^3^, generating another 2,438 sequences. This method entailed segmenting longer protein sequences into shorter fragments, adhering to the length distribution characteristic of our known sequences.

For the hemolysis classifier, we had a fortuitous situation where the numbers of non-hemolytic and hemolytic peptides were relatively balanced. As such, we deemed it unnecessary to create artificial sequences. Instead, we directly leveraged the existing set of peptides to train the hemolysis classifier, ensuring authenticity in the model's learning process.

### *C. acne*s associated AMP dataset

To pinpoint AMPs that target *C. acnes* and its closely related species, we embarked on the phylogenetic analysis. Our approach was as follows:

1. Data Acquisition: We began by downloading the names of all bacterial species cataloged in the DBAASP database.
2. 16S rDNA Sequence Retrieval: For each species name, we sourced its corresponding 16S rDNA sequence from the NCBI database. This ribosomal RNA sequence is universally present in bacteria and serves as a fundamental marker in microbial phylogenetics.
3. Phylogenetic Tree Construction: Using the retrieved 16S rDNA sequences, we utilized the MEGA software to construct a comprehensive phylogenetic tree. This tree visually represents evolutionary relationships, allowing us to discern which species are closely related to *C. acnes*.
4. Selection of Closely Related Species: From the constructed phylogenetic tree, we pinpointed 28 species that displayed close evolutionary ties with *C. acnes*.
5. Extraction of Relevant AMPs: With our selected species in hand, we sifted through the DBAASP database to identify AMPs that exhibited inhibitory activity against these species. Filtering further for peptides that were both active and non-hemolytic, we amassed a collection of 653 AMPs. These were designated as AMPs intrinsically associated with *C. acnes*.

The end result of this rigorous process was a curated set of AMPs tailored to target *C. acnes* and its closely related bacterial species, providing a focused dataset for subsequent analysis and experimentation.

We used python 3.10, pandas 1.5.0, and MEGA 11.0.13 to do data curations above.

## Comparison with Conventional Methods

We emphasized that the goal of this paper is not to provide AMP prediction models that outperform existing ones. Rather, the goal is to build a pipeline with comparable accuracies and selection strategies to design novel and potent peptides anti specific strains, such as C. acnes. Note that comparing different AMP prediction models is non-trivial, as different models vary widely by training AMP dataset size and data curation criteria, and some models are not open to public.

However, we still benchmarked our peptides against the AntiBP1^4^ prediction tool, which is recognized in the field, as shown in Table 1. Notably, our strongest AMP candidates, AMP-9 and AMP-12, were incorrectly predicted as non-AMP by both support vector machine (SVM) and artificial neural network (ANN) models within AntiBP1, despite their demonstrated efficacy (2-8 μg/mL MIC). Moreover, the prediction tool could only predict peptides with lengths more than 15 amino acids, so it failed to predict our AMP-31, AMP-33 and AMP-38, which are of 14 amino acids. This underscores the potential of our model to identify potent AMPs that other models may overlook.

## Supplementary Figures 1 to 3


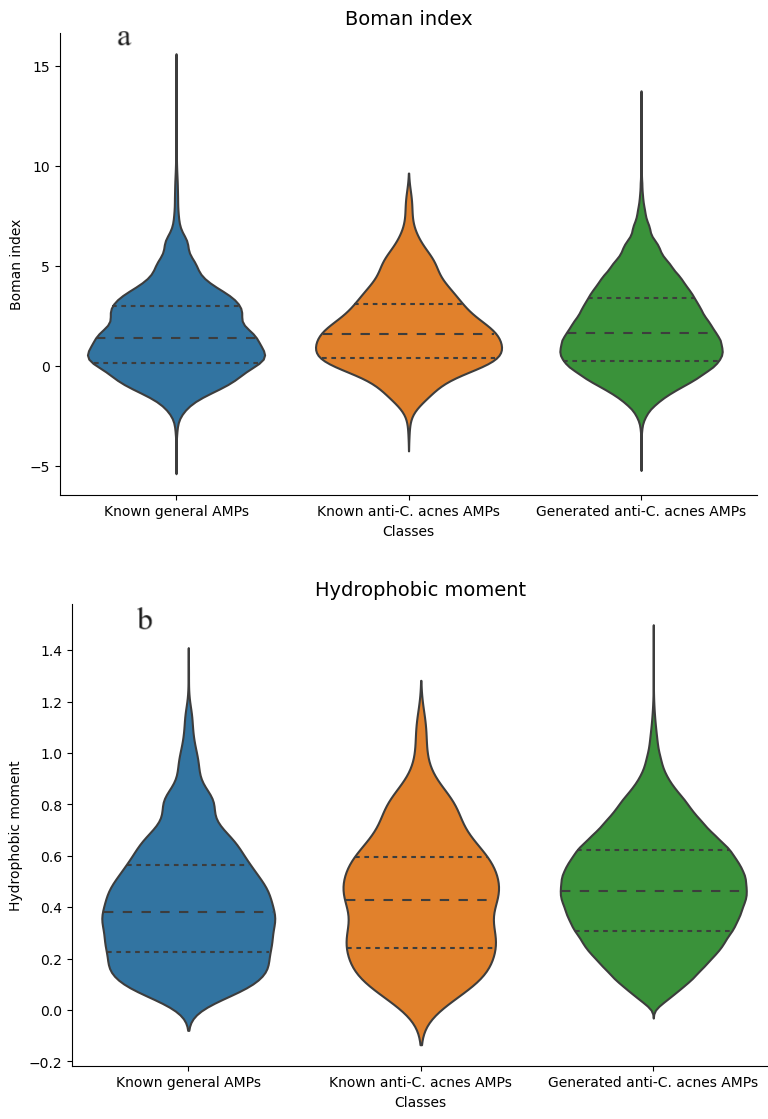


**Figure 1.** Comparison of physicochemical properties. (a) Boman index, (b) hydrophobic moment. "Known general AMPs" representing the 8884 known active AMPs in DBAASP, "Known anti-C. *acnes* AMPs" representing the 653 active and non-hemolytic *C. acnes*-related AMPs, and "Generated anti-*C. acnes* AMPs" representing the 660,000 sequences generated in this study.


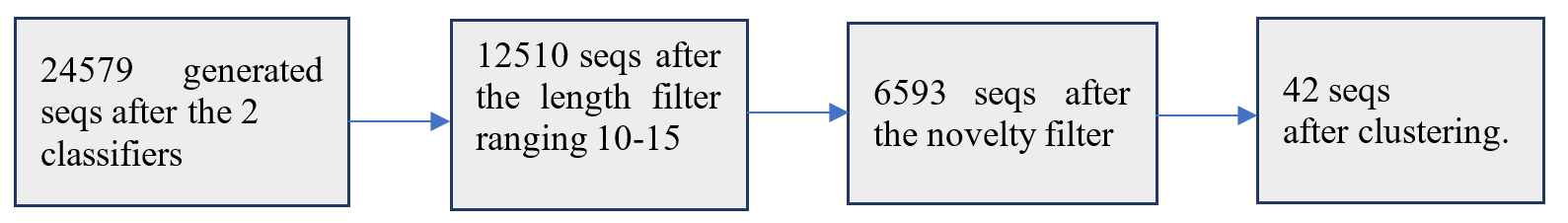


**Figure 2.** The length and novelty filtering pipeline and results.


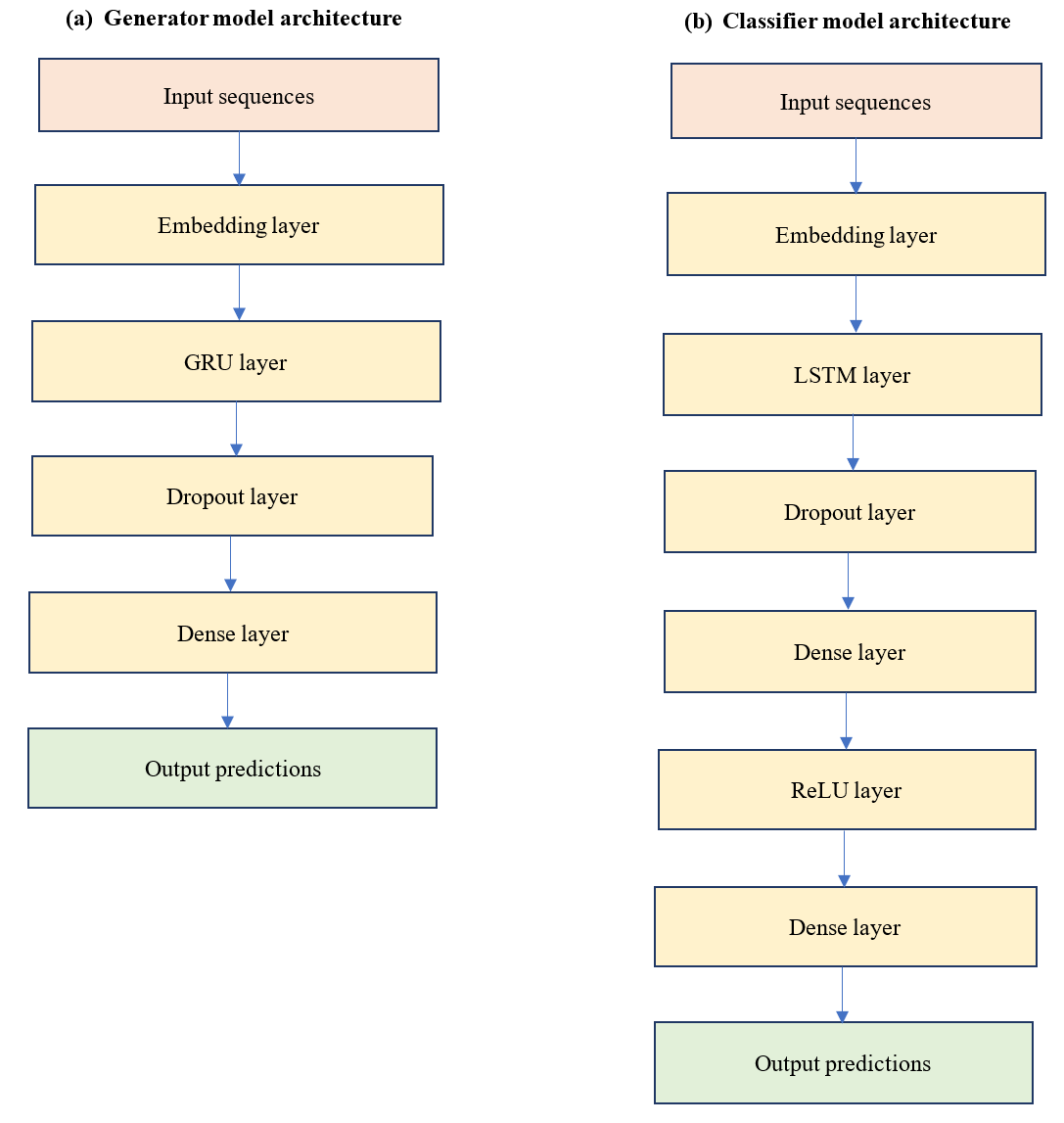


**Figure 3.** AI model structures. (a) The generator model structure, (b) the classifier model structure.

## Supplementary Tables in separate spreadsheet files (.xlsx format)

Supplementary Table 1. Prediction results on the identified AMPs using AntiBP1

Supplementary Table 2. Amino acid composition statistical analysis.

Supplementary Table 3. Physicochemical properties of designed peptides.

Supplementary Table 4. Total peptide sequences of in vitro experiments.

Supplementary Table 5. All the in vitro experiment data.

## References

1. DBAASP. DBAASP dataset. https://dbaasp.org/statistics?page=general-statistics (2023).

2. Capecchi, A. *et al.* Machine learning designs non-hemolytic antimicrobial peptides. *Chem Sci* **12**, 9221–9232 (2021).

3. Bateman, A. UniProt: a worldwide hub of protein knowledge. *Nucleic Acids Res* **47**, D506–D515 (2019).

4. Lata, S., Sharma, B. K. & Raghava, G. P. S. Analysis and prediction of antibacterial peptides. *BMC Bioinformatics* **8**, 1–10 (2007).
